# Spatiotemporal and molecular epidemiology of cutaneous leishmaniasis in Libya

**DOI:** 10.1371/journal.pntd.0005873

**Published:** 2017-09-07

**Authors:** Ahmad Amro, Hamida Al-Dwibe, Aisha Gashout, Olga Moskalenko, Marlena Galafin, Omar Hamarsheh, Marcus Frohme, Anja Jaeschke, Gabriele Schönian, Katrin Kuhls

**Affiliations:** 1 Faculty of Pharmacy, Al-Quds University, Abu-Dies, Jerusalem, Palestine; 2 Faculty of Medicine, Dermatology Department, University of Tripoli, Tripoli, Libya; 3 Faculty of Medical Technology—Pathology Department, University of Tripoli, Tripoli, Libya; 4 Molecular Biotechnology and Functional Genomics Department, Technical University of Applied Sciences Wildau, Wildau, Germany; 5 Department of Biological Sciences, Faculty of Science and Technology, Al-Quds University, Abu-Dies, Jerusalem, Palestine; 6 Department of Biogeography, University of Bayreuth, Bayreuth, Germany; 7 Institut für Mikrobiologie und Hygiene, Charité Universitätsmedizin Berlin, Berlin, Germany; Institut de Recherche pour le Développement, FRANCE

## Abstract

**Background:**

Cutaneous leishmaniasis (CL) is a major public health problem in Libya. In this paper, we describe the eco-epidemiological parameters of CL during the armed conflict period from January 2011 till December 2012. Current spatiotemporal distributions of CL cases were explored and projected to the future using a correlative modelling approach. In addition the present results were compared with our previous data obtained for the time period 1995–2008.

**Methodology/Principal findings:**

We investigated 312 CL patients who presented to the Dermatology Department at the Tripoli Central Hospital and came from 81 endemic areas distributed in 10 districts. The patients presented with typical localized lesions which appeared commonly on the face, arms and legs. Molecular identification of parasites by a PCR-RFLP approach targeting the ITS1 region of the rDNA was successful for 81 patients with two causative species identified: *L*. *major* and *L*. *tropica* comprised 59 (72.8%) and 22 (27.2%) cases, respectively. Around 77.3% of *L*. *tropica* CL and 57.7% of *L*. *major* CL caused single lesions. Five CL patients among our data set were seropositive for HIV. *L*. *tropica* was found mainly in three districts, Murqub (27.3%), Jabal al Gharbi (27.3%) and Misrata (13.7%) while *L*. *major* was found in two districts, in Jabal al Gharbi (61%) and Jafara (20.3%). Seasonal occurrence of CL cases showed that most cases (74.2%) admitted to the hospital between November and March, *L*. *major* cases from November till January (69.4%), and *L*. *tropica* cases mainly in January and February (41%). Two risk factors were identified for the two species; the presence of previously infected household members, and the presence of rodents and sandflies in patient’s neighborhoods. Spatiotemporal projections using correlative distribution models based on current case data and climatic conditions showed that coastal regions have a higher level of risk due to more favourable conditions for the transmitting vectors.

**Conclusion:**

Future projection of CL until 2060 showed a trend of increasing incidence of CL in the north-western part of Libya, a spread along the coastal region and a possible emergence of new endemics in the north-eastern districts of Libya. These results should be considered for control programs to prevent the emergence of new endemic areas taking also into consideration changes in socio-economical factors such as migration, conflicts, urbanization, land use and access to health care.

## Author summary

Cutaneous leishmaniasis (CL) is a skin infection caused by a single-celled parasite that is transmitted by the bite of a phlebotomine sandfly. CL is the most common form of leishmaniasis characterized by localized lesions in the skin and mucous membranes. The disease is prevalent in all countries around the Mediterranean Basin. In this paper, we describe spatiotemporal and eco-epidemiological parameters of CL in Libya. Moreover, we explored current spatiotemporal distributions of CL cases and explored the future projection of the disease. Our study indicates the presence of higher risk of CL in the coastal regions of Libya. Future projection until 2060 showed a trend of increasing incidence of CL in the north-western part of Libya, a spread along the coastal region and a possible emergence of new endemics in the north-eastern districts of Libya. These scenarios should be considered by health authorities in order to develop appropriate intervention strategies and plan effective control programs.

## Introduction

*Leishmaniasis* is a group of vector–borne diseases caused by obligatory intracellular parasitic protozoans belonging to the genus *Leishmania*. The clinical manifestations range from cutaneous and muco-cutaneous leishmaniasis (CL and MCL) which are characterized by localized lesions in the skin and mucous membranes, to visceral leishmaniasis (VL) which is the most severe form and mostly fatal in developing countries if untreated [1]. These manifestations (dermotropic and viscerotropic) depend on the causative *Leishmania* species and genotypes, the geographical origin of the cases [2–4], and the immune response of the infected host [5].

CL is prevalent in more than 70 countries throughout Africa, Asia, South Europe, and North and South America [6]. The countries with the highest number of reported cases are Algeria, Brazil, Iran, Syria, Afghanistan, Pakistan, Tunisia and Peru [7]. All countries around the Mediterranean Sea are endemic for CL, including North Africa from Morocco to Egypt [7,8] where CL transmission has been increasing since the 1980s and thousands of cases are reported every year [8]. However, underreporting of CL is a serious problem in many endemic countries [7,9]. Four species of *Leishmania* were identified as causative agents of CL in the Old World. *Leishmania major* is most frequent and causes more than 90% of the registered cases in Algeria, Tunisia and Libya. *Leishmania tropica* (syn. *L*. *killicki*) is more prevalent in Morocco causing 30–40% of the CL cases in some districts [10]. *Leishmania aethiopica* is found exclusively in Africa and is considered as the main causative agent of CL in Ethiopia and Kenya [7,11]. Less frequently, CL can be also due to *L*. *infantum*, the well-known agent of VL in the Mediterranean region [3,7–9,12–14]. The three species *L*. *major*, *L*. *tropica* and *L*. *infantum* are sympatric in all North African CL endemic countries, although they differ in modes of transmission, zoonotic vs. anthroponotic [8,15], reservoir hosts [8,15–19], *Phlebotomus* sandfly vectors [8,20], and eco-epidemiological characteristics [7,8,12,21]. This polymorphic presentation of CL is quite complex and challenges the national prevention and control programs established by many North African countries. Different measures applied for the containment of vectors and animal hosts failed to stop the spread of CL [22]. Moreover, no vaccine for CL is available and treatment failures are reported in many endemic countries. This has consequently increased CL burden on human health and societies [23,24].

In Libya, CL is distributed nearly exclusively in north-western districts where *L*. *major* is the dominant causative species followed by *L*. *tropica* [12], while *L*. *infantum* is hypo-endemic and restricted to young people under 20 years old [14]. In 2009 an emerging focus was reported from Sirte in the northern center of the country [25]. The first case of CL caused by *L*. *tropica* was documented from the district Misrata (Beni Walid) in 2006 [26]. In 2012 molecular typing could prove for the first time the occurrence of this species in the districts Al Jabal Al Gharbi, Misrata,Murqub [12], Nuqat al Khams, Zawiya, and Jafara [26], and additionally in Nalut [14]. Four years later, the circulation of *L*. *tropica* was confirmed by molecular methods also in Tripoli and Al Jabal Al Gharbi (Zantan and Gharyan) [27]. The most southern proven occurrence of *L*. *tropica* was in Wadi Al Hayaa [12]. *Leishmania major* was found mainly in rural regions, *L*. *tropica* rather in urban areas [27]. Seasonal distribution of CL was documented and has shown a peak during November-February [12,14]. The reservoirs of *L*. *major* in Libya are *Psammomys obesus* (sand rat) and *Meriones spp*. (gerbils and jirds) with *Phlebotomus papatasi* as the transmitting vector [7,28]. In Misrata district *Leishmania* DNA was detected in *Ph*. *papatasi* and *Ph*. *longicuspis* sandfly species, however the causative species remained undetermined [29]. Parasite life cycles, transmitting vectors and reservoir hosts of CL caused by *L*. *tropica* were not yet investigated, usually *L*. *tropica* is associated with anthroponotic transmission, however, the existence of putative animal reservoirs is also discussed [8,30].

Diagnosis of leishmaniasis in Libya is still based on evaluation of the clinical picture and microscopy of stained skin biopsies and only recently first molecular typing studies were published [12,14,27].

Since February 2011, Libya is living in an armed conflict (Libyan revolution) that led to the collapse of the old regime in October 2011 and its replacement by the National Transitional Council which declared the end of the war and the liberation of Libya. However, sporadic clashes continued across the country and fighting broke out again in January 2012 descending Libya into an ongoing low-level civil war. This situation has influenced all life aspects in Libya including migration of civilians and poorer coordination between humanitarian agencies. It also led to the aggravation of the health care system and the disruption of national disease control programs, thus accumulating risk factors that contributed to the spread and the proliferation of CL.

In this study, we continue our survey of eco-epidemiological parameters of CL in Libya that started with a general analysis and molecular identification of the causative agents of cases that occurred between 1995 and 2008 [12]. Here we analyze CL cases that were recorded during the period of the armed conflict from January 2011 till December 2012 and we investigated demographic characteristics of all cases, the clinical evaluation of patients and molecular characterization of parasites. We explored the spatial distribution of CL cases and projected future distributions based on climate change scenarios that could affect occurrence and/or density of the sandfly vectors to assess the expansion potential and risk factors of the disease in Libya. Moreover, we are discussing the activities of *Leishmania* national control program (LNCP) in Libya and the consequences of the armed conflict on CL surveillance and control. We compared the results of this study with our previous study done in 1995–2008 [12].

## Materials and methods

### Cutaneous leishmaniasis patients

During the period between January 2011 and December 2012, we investigated all patients (in total 312) presenting to the Dermatology Department in the Tripoli Central Hospital (TCH) with skin lesions. These patients were referred for clinical evaluation and diagnosis for CL by direct smear microscopy. Patient’s skin lesion and adjacent areas were cleaned with 70% ethanol for sterilization. Tissue biopsies were taken using scalpel blades. Small incisions were made in the lesion’s margin to remove and pick up skin tissues which were then smeared on two clean glass microscope slides. One slide was stained with Wright’s Giemsa stain and the other kept for DNA extraction. Stained slides were examined for the presence of *Leishmania* amastigote bodies by light microscopy at 400 x magnification.

Demographic data for spatial and epidemiological analysis was collected for each patient including date of infection, age, gender and place of residence. Clinical presentation including number and location of lesions and treatment response were documented. Moreover, patients were questioned about household members previously diagnosed with CL, and the presence of rodents and sandflies in their neighborhood to assess their risk.

### DNA extraction and molecular characterization of Leishmania

All slides were kept at 4°C until being transferred and analyzed in Al-Quds University in Palestine and Technical University of Applied Sciences Wildau, Germany. 250 μl of lysis buffer composed of 50 mM NaCl, 50 mM Tris, 10 mM EDTA, pH 7.4 were added to each glass slide. Materials were then scraped from slides by using filter tips and transferred into 1.5ml Eppendorf tubes. Triton X-100 and Proteinase K were added to final concentrations of 1% and 200 μg/ml, respectively and the tubes were incubated overnight at 60°C to accomplish cell lysis. The phenol-chloroform extraction method was applied to extract DNA from lysates as described previously [31–33]. DNA pellets were dried using a speed vacuum dryer and re-dissolved in 40 μl TE buffer (1mM EDTA and 10mM Tris, pH 7.5). Additional DNA purification was carried out using a DNA purification kit (Qiagen). The DNA was then kept at -20°C until use.

A PCR targeting the ribosomal internal transcribed spacer 1 (ITS1) of *Leishmania* was performed using the primer pair LITSR (5`-CTGGATCATTTTCCGATG-3´) and L5.8S (5´-TGATACCACTTATCGCACTT-3´) [34] including also negative and positive controls as described elsewhere [12]. This spacer is polymorphic among different *Leishmania* species which can be distinguished by digesting the ITS-1 amplicon with the restriction enzyme *HaeIII* [35,36]. WHO reference strains for *L*. *major* (MHOM/SU/1973/5ASKH), *L*. *tropica* (MHOM/SU/1974/SAF-K27) and *L*. *infantum* (MHOM/ES/1993/PM1) were included as controls and for comparison in each experiment. RFLP products were separated by electrophoresis in 2% Metaphor gels in 1xTAE buffer or alternatively by using the Fragment Analyzer (Advanced Analytical Technologies, Inc.).

### Spatiotemporal analysis and spread potential

Correlative distribution modelling was performed with the database of CL cases confirmed by microscopy for the time periods 1995–2008 (450 cases), 2011–2012 (312 cases) and for the whole period 1995–2012 (762 cases) as occurrence data. Conclusions were made based on these data regarding the distribution of climatically suitable areas for the disease transmission based on sandfly biology.

The geographical location (latitude, longitude) was obtained using GeoLocator (http://tools.freeside.sk/geolocator/) (version 1.35).

Based on a literature review of ecological and environmental factors affecting sandfly distribution [37,38], the following eight of the 19 bioclimatic variables of Worldclim (http://www.worldclim.org/bioclim) [39] were selected for the current and future projections: BIO1—Annual Mean Temperature, BIO2—Mean Diurnal Range (mean of monthly (max temp—min temp)), BIO4—Temperature Seasonality (standard deviation *100), BIO7—Temperature Annual Range (BIO5-BIO6), BIO10—Mean Temperature of Warmest Quarter, BIO11—Mean Temperature of Coldest Quarter, BIO12—Annual Precipitation, and BIO15—Precipitation Seasonality (Coefficient of Variation). All climate data have spatial resolution of 5 arc-minutes (approximately 10 km).

To model the distribution of CL four modelling algorithms were applied: GLM (Generalized Linear Model) [40], GBM (Generalized Boosted Model) [41] and RF (Random Forest) [42] included in the biomod2 R-Package version 3.3–7 (https://CRAN.R-project.org/package=biomod2) [43] and MAXENT (Maximum-Entropy-Modelling) [44] Ensemble modelling [45,46] was performed using these four algorithms with each algorithm having the same weighting (25%). The available data from 1995–2008, 2011–2012 and for the whole period (1995–2012) were used to calculate the current and the future projections (2041–2060) for CL. For future projections, the climate model mpi-esm-lr (http://ccafs-climate.org/data_spatial_downscaling/) and the emission scenario RCP 4.5 were used. RCP 4.5 is an intermediate scenario expecting a raise in the global mean surface temperature between 1.1°C and 2.6°C until the end of the century [47]. Mapping of the derived projections was done using GIS software (QGIS 2.8.4-Wien, http://www.qgis.org/de/site/).

### Ethical considerations

The study design and protocols were revised and approved by the Research Ethics Committee in the Faculty of Medicine, University of Tripoli, Libya. Study objectives and procedures were explained to each patient (respectively parent). Informed consent was obtained in written form from each participant (or the parents/guardians on behalf of the children under the age of 15). All samples were anonymized and given special codes which have been used for laboratory experiments and data analysis of each sample.

## Results

**Fig 1:**
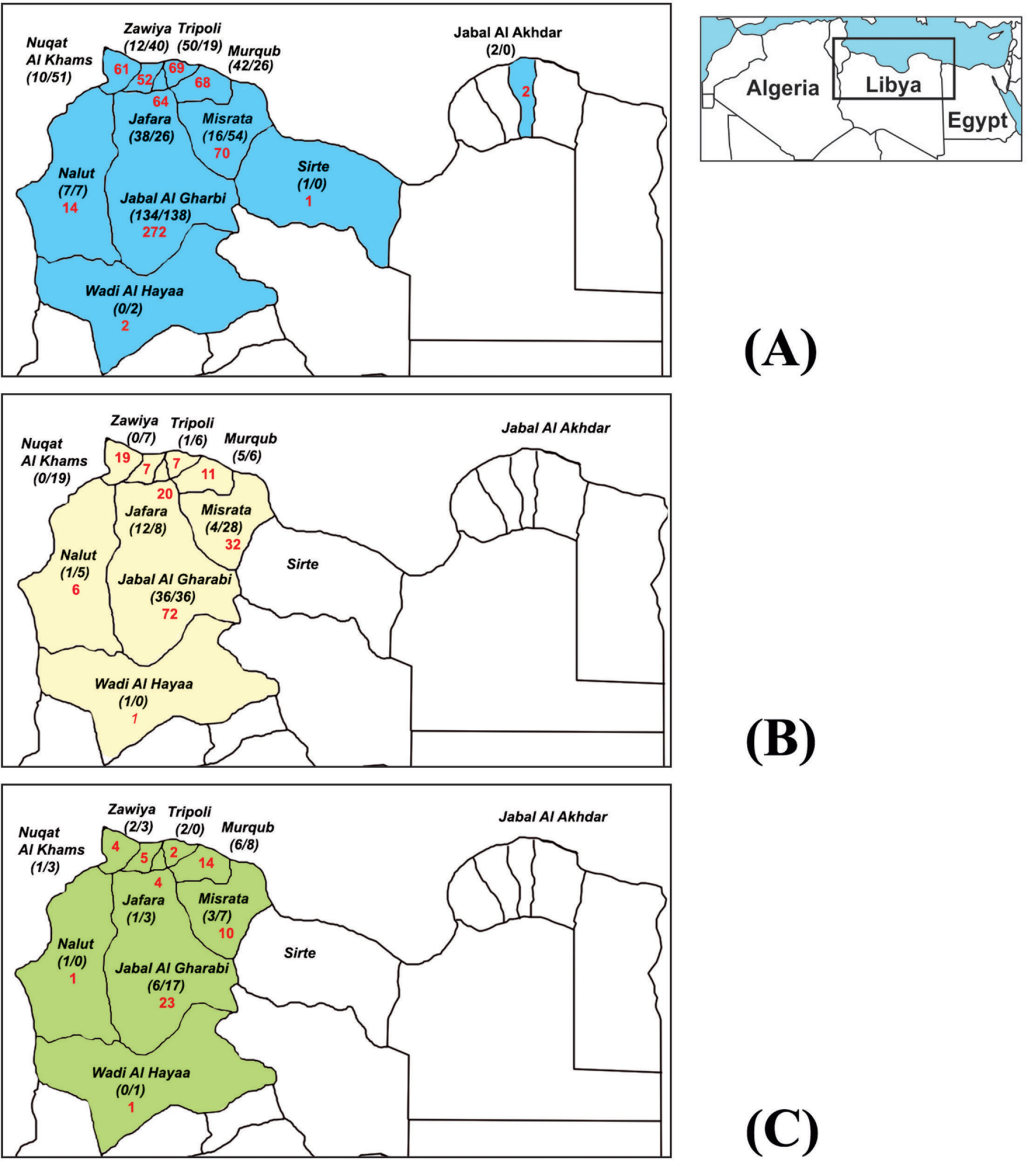
**Geographical origin of cutaneous leishmaniasis cases collected during the two time periods 2011–2012 and 1995–2008: (A)** including all microscopically confirmed cases, **(B)** showing all cases caused by *L*. *major*. **(C)** showing all cases caused by *L*. *tropica*. Red numbers are indicating the overall number for both time periods. The first number in brackets indicates the number for 2011–2012, the second one for 1995–2008. Data for 1995–2008 are based on a previous study [12]. The maps have been developed based on Landsat Look Viewer which is available free of charge on the internet under the following link https://landsatlook.usgs.gov/viewer.html.

During the study period, 312 patients with skin lesions were investigated for CL infections at the Dermatology Department of Tripoli Central Hospital (TCH). The patients came from 81 endemic areas located in 10 districts mainly in Northwest Libya. 134 patients were from Jabal al Gharbi, 50 from Tripoli District, 38 from Jafara, 42 from Murqub, 10 from Nuqat al Khams, 12 from Zawiya, 16 from Misrata, two from Jabal al Akhdar, 7 from Nalut and one from Sirte District (Fig 1A, Table 1).

**Table 1:** Distribution of cutaneous leishmaniasis cases per district, method of identification, causative *Leishmania* species and study period.

	2011–2012						1995–2008^1^					
	Microscopy		PCR-RFLP				Microscopy		PCR-RFLP			
district	Cases	%	*L*. *major %*		*L*. *tropica*	%	Cases	%	*L*. *major*	%	*L*. *tropica*	*%*
Nuqat al Khams	10	*3*.*2*	0	*0*	1	*4*.*5*	51	*11*.*3*	19	*12*.*8*	3	*6*.*4*
Zawiya	12	*3*.*9*	0	*0*	2	*9*.*1*	40	*8*.*9*	7	*4*.*7*	3	*6*.*4*
Jafara	38	*12*.*2*	12	*20*.*3*	1	*4*.*5*	26	*5*.*8*	8	*5*.*4*	3	*6*.*4*
Tripoli	50	*16*	1	*1*.*7*	2	*9*.*1*	19	*4*.*2*	6	*4*.*1*	0	*0*
Murqub	42	*13*.*5*	5	*8*.*5*	6	*27*.*3*	26	*5*.*8*	6	*4*.*1*	8	*17*.*0*
Misrata	16	*5*.*1*	4	*6*.*8*	3	*13*.*7*	54	*12*.*0*	28	*18*.*9*	7	*14*.*9*
Sirte	1	*0*.*3*	0	*0*	0	*0*	0	*0*.*0*	0	*0*	0	*0*
Jabal al Akhdar	2	*0*.*6*	0	*0*	0	*0*	0	*0*.*0*	0	*0*	0	*0*
Nalut	7	*2*.*2*	1	*1*.*7*	1	*4*.*5*	7	*1*.*6*	5	*3*.*4*	0	*0*
Jabal al Gharbi	134	*43*	36	*61*.*0*	6	*27*.*3*	138	*30*.*7*	36	*24*.*3*	17	*36*.*2*
Wadi Al Hayaa	0	*0*	0	*0*	0	*0*	2	*0*.*4*	1	*0*.*7*	1	*2*.*1*
Missing	0	*0*	0	*0*	0	*0*	87	*19*.*3*	32	*21*.*6*	5	*10*.*6*
**Total**	**312**	***100***	**59**	***100***	**22**	***100***	**450**	***100***	**148**	***100***	**47**	***100***

^1^ Data set published in Amro et al. (2012) [12], including all positive cases identified by microscopy and PCR-RFLP that were stored in the archive of the Libyan National Centre for Infectious Diseases and Control (LNCIDC) between 1995 and 2008.

**Fig 2:**
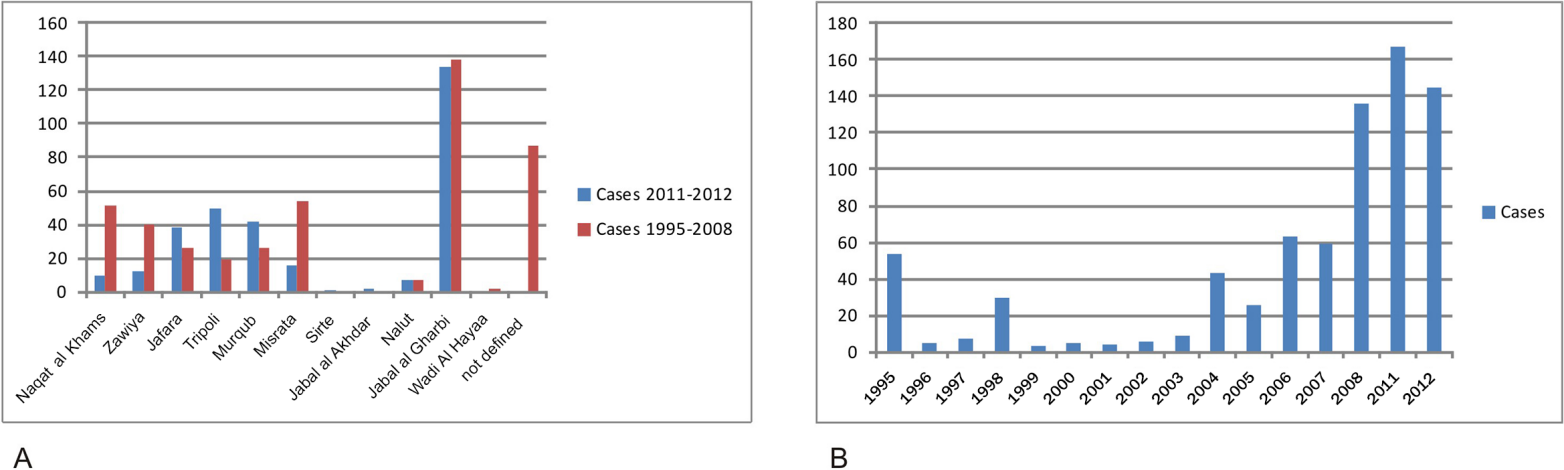
Distribution of cutaneous leishmaniasis cases in Libya by district and year of infection. **(A)** Comparison of the number of microscopically confirmed cases found in the respective districts for the time periods 2011–2012 (all cases registered in the Tripoli Central Hospital) and 1995–2008 (all cases stored in the archive of the Libyan National Centre for Infectious Diseases and Control (LNCIDC)) [12]. **(B)** Progression in time of the number of the collected and verified cases including both time periods.

The case numbers collected from the respective districts for the present time period and between 1995 and 2008 in our previous study that included all microscopically confirmed cases stored in the archive of the Libyan National Centre for Infectious Diseases and Control (LNCIDC) [12] are compared in Fig 2A, Table 1 and Fig 1A–1C. The progression in time of the collected and verified cases including both time periods is shown in Fig 2B.

The district with the highest number of cases was for both time periods Jabal al Gharbi, followed by Misrata, Nuqat al Khams and Zawiya in 1995–2008, and Tripoli, Murqub and Jafara in 2011–2012 (however this could be attributed to the sampling of cases in different hospitals). An increase of the overall number of cases per year was observed from 2004 on with the highest number of cases in 2008 till 2012. This high number in 2011 and 2012 is striking taking into consideration, that those cases were collected only in a single hospital.

*Leishmania* amastigotes were seen in all Giemsa stained slides by light microscopy, however no records were available concerning the parasitic load of the respective samples. The second copy of each slide was subjected to DNA extraction and species identification using PCR-RFLP. Amplification of the ITS1 region was successful for 81 patients showing the ~300 bp band of the PCR product characteristic for the *Leishmania* genus [35,36]. The digestion of this amplicon with restriction endonuclease *Hae*III revealed two causative species when compared to RFLP profiles of reference strains. *Leishmania major* was detected in 59 of the 81 samples (72.8%) and *L*. *tropica* in 22 (27.2%) (Table 1). Two of the latter cases were brothers from Tarhuna in Murqub District.

**Fig 3:**
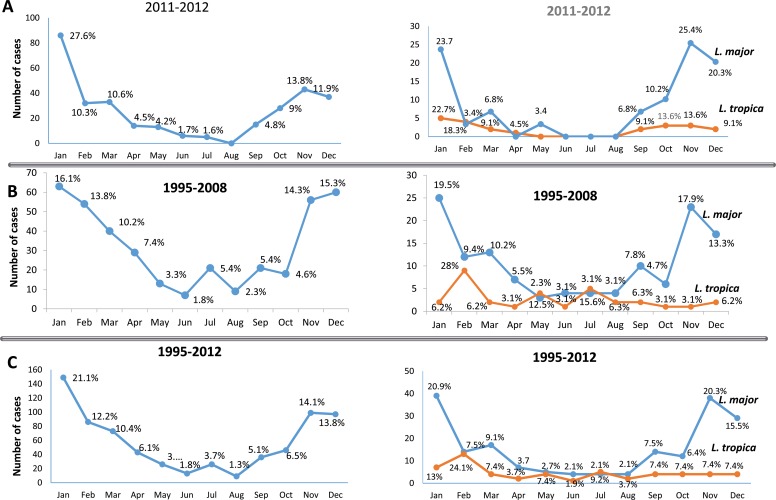
Per-month distribution of Libyan cutaneous leishmaniasis (CL) cases (left charts) and seasonal distribution of CL cases caused by *L*.*major* and *L*.*tropica* (right charts). **(A)** distribution of CL cases collected during 2011–2012, **(B)** distribution of CL cases collected during 1995–2008 as published elsewhere [12], **(C)** overall distribution 1995–2012.

About 74.2% of the CL cases collected during this study were recorded from November to March with the highest peak in January (27.6%), and a decline between April and October (Fig 3A). The same trend of seasonality was observed in our study during 1995–2008 when the highest peak being in January (Fig 3B) [12]. The percentages of cases per month were similar for both time periods (e.g. April-October < 9%). Noticeable was the very high number of cases in January in the time period 2011–2012. Analysis of the whole data set collected during 1995–2012 showed that 71.6% of cases were recorded during the months November-March with a maximum of cases of 21.1% in January (Fig 3C). Seasonal distribution of causative species showed a peak of *L*. *major* from November–January (69.4%), while *L*. *tropica* cases peaked in January and February (41%) and no cases (with one exception in April) between April and August (Fig 3A). In the time period 1995–2008 the majority of cases were recorded in February, however there was a continuous occurrence of several cases in the summer months (Fig 3B). The seasonality analysis of cases of the combined time period (1995–2012) is shown in Fig 3C: *L*. *tropica* is recorded with the same frequency and in low numbers all over the year with a maximum of cases in January and February (13% and 24.1%, respectively); *L*. *major* cases were recorded mainly from November till January (56.7%).

The male: female ratio in the present study was 1.29: 1. The average age at the onset of disease was 30 years ranging from 6 months to 85 years (median = 28).

**Fig 4:**
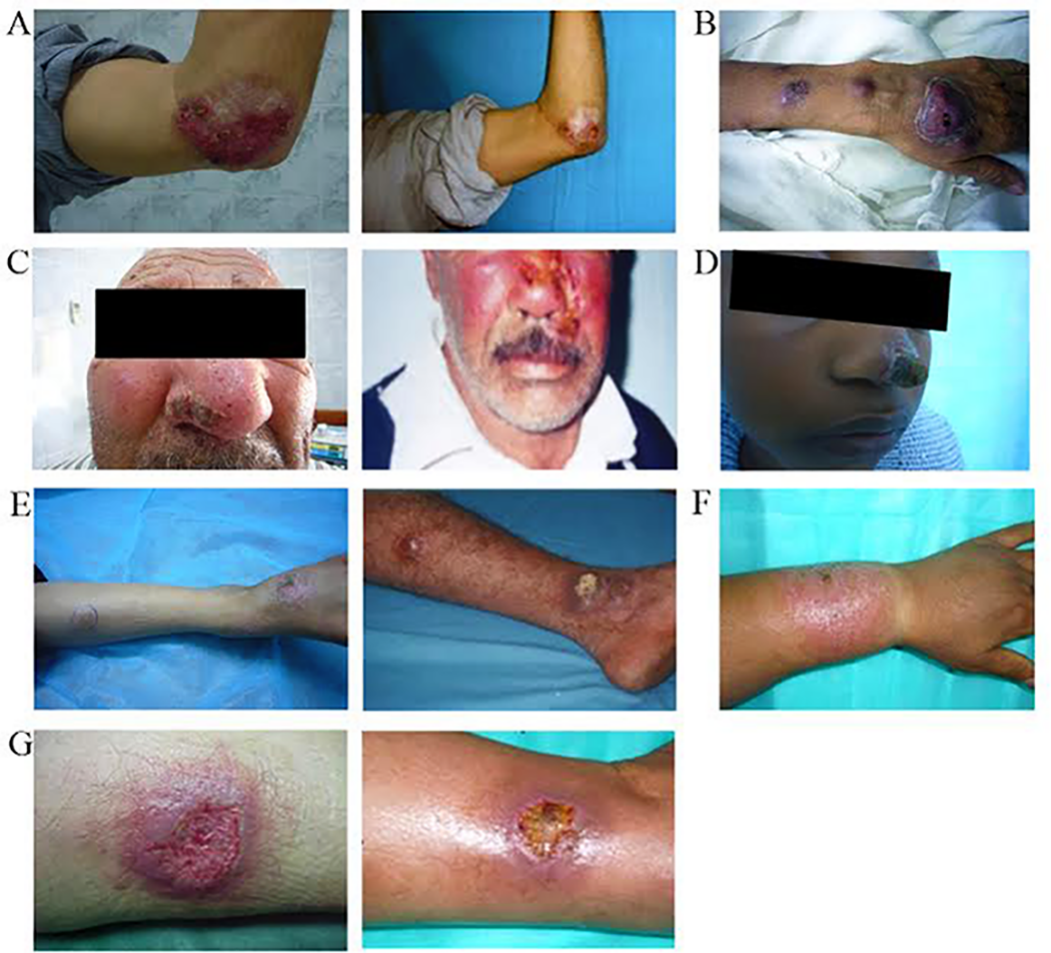
Clinical presentation of cutaneous leishmaniasis. **(A)** Leishmaniasis recidivans (lupoid) activated after 2 years from clinical cure. **(B)** Sporotrichoid type of CL. **(C)** Cold cellulitis like CL. **(D)** Nodulo-crusted CL. **(E)** CL with nodular lymphangitis. **(F)** Large nodular type. **(G)** Ulcerated type CL.

The 312 patients included in this study presented with single (170 cases, 54%) or multiple lesions (142 cases, 46%), respectively.77.3% of *L*. *tropica* CL lesions were single compared to 57.7% of *L*. *major* lesions. The lesions appeared typically on exposed parts of the body, most commonly on face and extremities (arms, legs and feet). Table 2 shows per-species distribution of CL cases with site and frequency of lesions. These lesions were usually painless and evolved from papules to nodular plaques to ulcerative lesions with depressed centers, raised borders and variable surrounding indurations. Some of the lesions were covered by crust in the central part and became painful especially after secondary bacterial infection. However, nodular lymphangitis was noticed in many cases. Fig 4 summarizes the clinical symptoms of CL in our patient cohort. Interestingly, one patient from Zuwarah (Nuqat Al Khams district) had a leishmaniasis recidivans (lupoid) with a recurrence of lesions two years after successful treatment (Fig 4A).

**Table 2:** Distribution of cutaneous leishmaniasis cases collected in the present study (2011–2012) with single and multiple lesions and site of lesions according to the causative *Leishmania* species.

	Single lesions		Multiple lesion		Total
Site of lesions	Face	Extremities	Face and Extremities	Extremities	
*L*. *major*	8 (13.6%)	26 (44.1%)	3 (5%)	22 (37.3%)	59
*L*. *tropica*	7 (31.8%)	10 (45.5%)	4 (18.2%)	1 (4.5%)	22
Total	15 (18.5%)	36 (44.4%)	7 (8.7%)	23 (28.4%)	81

All patients were treated successfully with intramuscular sodium stibogluconate (Pentostam) pentavalent antimony applying 20 mg Sb5 per kg body weight daily for 21 days. No changes in treatment regime and response were reported in this patient cohort. Five CL patients among our studied cases were seropositive for HIV. All of them were males between 22 and 47 years old with an average age of 37.4 years. They were treated efficiently with Pentostam and no treatment failure was reported.

Patients were surveyed about the occurrence of previous CL infections of household members, and the presence of rodents and sandflies in their neighborhood. One hundred and sixty-eight patients (53.8%) reported the presence of at least one previously infected household member. The presence of sandflies or rodents or both was reported by 230 patients (73.7%), 56 patients (17.9%) did not recognize their presence and 26 patients (8.3%) did not provide data.

**Fig 5:**
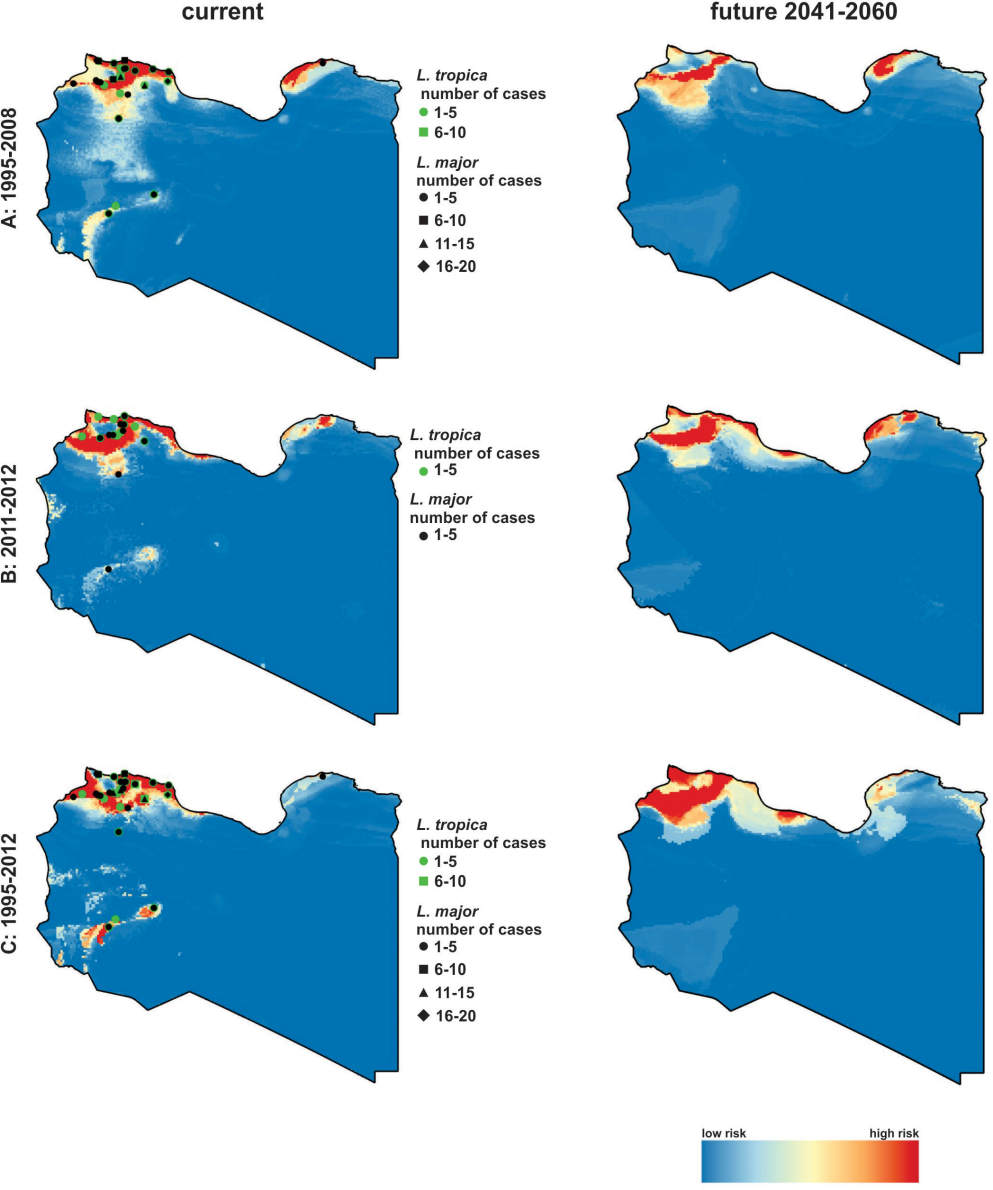
Spatiotemporal analysis of the Libyan CL. **(A)**Spatiotemporal analysis of the Libyan datasets of microscopically confirmed cases of (CL) of the time periods 1995–2008 investigated in a previous study [12], **(B)** Spatiotemporal analysis of the present study 2011–2012. **(C)** The combined datasets for 1995–2012. Modelled current and future climatic suitabilities for CL are shown for the respective time periods. Projections for the presence (left line) and the future (right line) are shown. Future projections were calculated for the years 2041–2060 using the mpi-esm-lr climate model and the RCP 4.5 scenario. Molecular typing results were added to the presence projections (indicated as different symbols in the map), showing the occurrence and the number of *L*. *major* and *L*. *tropica* in the different regions of the country, however including only the respective subset of cases that were PCR positive.

Spatiotemporal analysis based on climatic conditions shows different climatic suitabilities for disease transmission in different parts of the country. This analysis included all microscopically confirmed clinical cases of CL, considering also the different periods of data collection. Fig 5 shows the present and potential future climatic suitabilities for CL cases for the period 1995–2008 (Fig 5A), 2011–2012 (Fig 5B) and the total period 1995–2012 (Fig 5C). The future projection was calculated for the years 2041–2060. Molecular typing results also were added to the presence projections (indicated as different symbols in the map), showing the occurrence and the number of *L*. *major* and *L*. *tropica* in the different regions of the country, however including only the respective subset of cases that were PCR positive.

## Discussion

Although several countries in North Africa, including Libya, have established national control and surveillance programs for containing the sandfly vector and recommending regimes for the treatment of CL infections, the disease continues to spread drastically. During the study period (2011–12), no changes were noticed in the geographical distribution of CL cases in Libya. Most cases came from the northwestern districts adjacent to the Mediterranean Sea that have climatic and environmental conditions favorable for the spread of CL. This distribution is consistent with previous studies done in Libya and in other North African countries [12,13,30,48–50]. Interestingly, *L*. *tropica* was found mainly in the three districts Jabal al Gharbi, Murqub and Misrata in both study periods. This distribution was confined to the urban areas of these districts compared to *L*. *major* which was found mainly in rural regions. These findings are in agreement with a recent study done by El-Badry et al. [27].

The regions with the highest climatic suitabilities for CL in the presence are found in the northwestern part of the country, where the main CL foci are located, including the districts Nuqat Al Khams, Zawiya, Jafara, Tripoli, Murqub and the northern parts of Nalut, Misrata and Jabal Al Gharbi (including the Djebel Neffoussa mountain chain) as well as the coastal region of Sirte. Further regions with a high climatic suitability are projected in the northeast of Libya in the districts Benghazi, Marj, Jabal Al Akhdar and Derna. Generally, there is high risk of CL occurrence due to climatic suitability for the vectors along almost the whole coastal region, with exception of the central coastline (Al Wahat). However, in Sirte only a very narrow band of the coastal region has a high suitability. Besides the coastal regions, also in the western central part of Libya regions are projected as suitable for the occurrence of CL. These regions are located in the districts Wadi Al Hayaa, Sabha and Wadi Al Shatii. The future projections indicate a stabilization of the northwest as a highly suitable region and an increase of the suitability in the northeast including also new parts in the most eastern coastal region (district Butnan). In general, the coastal regions that possess a high suitability and hence a high CL risk will expand slightly to the interior parts of the country. The foci in the central western regions of Libya are projected to disappear in the future and only the very North of the country will be at high risk of CL.

Spatiotemporal projections based on climatic conditions showed that coastal regions are projected to offer more favourable conditions for vectors transmitting *Leishmania* and therefore are at higher level of risk. Moreover, the future projection until 2060 indicates an increasing suitability for CL in the north-western regions, and a possible emergence of new endemics in the north-eastern districts of Libya (potential spread of CL along almost the whole coastal regions which are characterized by a Mediterranean climate). Conversely, a decreasing suitability can be expected in the central districts Wadi al Shatii, Sabha and Wadi al Hayaa because of the projected climate change with respect to the included bioclimatic variables. These changes include an increase of the temperature and a decrease of the humidity to a level that cannot be tolerated by the transmitting sand fly vectors. These results should be considered for monitoring and vector control programs to prevent the emergence of new endemic areas. The present conclusions are principally in line with recent findings from Samy et al. (2016) [51] where they projected the potential distribution of *L*. *major* along the coastline of Libya. Further analyses of the niche breadth of *L*. *major* revealed a preference for low elevations and maximum temperatures below 25–37°C [52–54].

The distribution of the vector depends on the environmental conditions. One of the most important factors in the survival, development, behaviour and activity of sandflies is the temperature [55]. Sandflies have a nocturnal activity, hence the daily maximum temperature is less important, but daily minimum temperature that normally occurs in the evening and at night is crucial. Another factor is the monthly activation, where the maximum and minimum temperature have a huge influence on survival, complete generation, quantity and fertility of sandflies as well as disease transmission. Humidity coming as rainfalls and condensation is important for the egg and larvae growth with a lesser role in adulthood. Therefore, as an independent factor in disease transmission it has less importance in adults but indirectly is very important in the pre-adult stages to reach the stage of a mature individual. However, in general humidity is less important than temperature. Climate change has the potential to increase the incidence and geographic range of leishmaniasis in Africa [56]. The incidence is associated with rainfall and minimum temperature. Expected climatic changes in North Africa are related to a likely increase in annual minimum and maximum temperature, where the minimum temperature is assumed to experience a greater increase. In contrast, precipitation has experienced a strong decrease over the last decades, especially in winter and early spring. Future projections expect a reduction in rainfall with seasonal drying and warming.

On the other hand, anthropogenic changes, e.g. the construction of dams, can change the temperature and moisture of soils and therewith the vegetation leading to changes in sandfly and rodent density and composition [56]. Hence, further research is needed due to the manifold underlying factors and their potentially contrasting effects.

When comparing demographic characteristics of patients in the present study, male to female ratio and patient’s average age and median did not show any significant differences and were constant with all studies done before in Libya [14,25,57–59].

Per-month distribution of CL cases during 2011–2012 was consistent with the seasonal occurrence of CL reported in Libya during 1995–2008 [12]. The only difference observed was the remarkable high number of cases in January in the present data set. The consistent seasonality was probably related to the sandfly’s biting season around the Mediterranean Sea which extends from May to October [54], different biting behaviors of transmitting sandfly species of *L*. *major* and *L*. *tropica* [60] and to the incubation period after infection which may last from one month for *L*. *major* to three months for *L*. *tropica* [58,61]. The clinical presentation of CL caused by *L*. *major* and *L*. *tropica* in Libya in terms of site and frequency of lesions is similar to that in other North African and Middle Eastern countries [30,62,63]. However, we reported one case of leishmaniasis recidivans in the Zuwarah District, which is a prolonged, relapsing form of CL that may persist for many years with a chronic and relapsing course. This form of leishmaniasis is not very common in Libya. *L*. *tropica* has been shown to be the causative species of this rare variant in the Old World. Molecular identification of the causative CL species was, however not possible for this patient.

Data about *Leishmania*-HIV co-infection in Libya is scarce and there was no report until 2014 [64]. Diagnosis of CL was based on microscopy and molecular identification of the causative species for this CL/HIV co-infection has failed unfortunately. An atypical presentation of CL was earlier reported from a Libyan HIV patient from Jabal al Gharbi who presented with contact dermatitis-like symptoms which was confirmed as localized CL due to the observation of *Leishmania* bodies in stained slit-skin smears after weeks of wrong treatment [64]. Thus, the development and implementation of diagnostic and control programs against CL-HIV coinfections in Libya is crucial.

The causative CL species in our sample collection was mostly *L*. *major*, and *L*. *tropica* was less prevalent. The clinical picture for all patients was compatible with the WHO manual for the management of CL cases without significant clinical differences depending on the infecting species [65]. The presence of *L*. *major* and *L*. *tropica* as causative agents of CL in Libya is consistent with all studies done previously in different CL foci in North Africa [8,12,21]. However, CL cases due to *L*. *infantum* were not detected though it has been described recently in the Nalut District [14]. This form of CL is probably not very common in Libya. *Leishmania infantum* is mainly causing VL in North African countries as also in general, the causative agent belongs to the MON-1 zymodeme and is distributed in the northern parts of these countries [8,65]. CL by *L*. *infantum* is rather rare, caused by dermotropic zymodemes as MON-24 and MON-80 and found mainly in the central parts of the countries as described in Algeria and Tunisia [2,3]. Molecular detection of *L*. *infantum* in CL lesions could be hindered by many factors, such as low intra-lesional parasitic load, DNA degradation and PCR inhibition. The same factors may also explain the low number of PCR positives in our sample set. Hence, DNA purification and the use of inhibition control should be mandatory for molecular diagnosis and species identification of *Leishmania* isolated from clinical samples. Another reason of the low number of PCR positives might be that we used duplicates of the clinical sample slides and in case of low parasitic load the outcome from DNA isolation might be too low. Some samples showed faint PCR bands, that were not suitable for RFLP analysis and hence were excluded from the study. The infecting agent affects treatment options, efficacy and duration as described by the WHO manual for case management of CL in the WHO Eastern Mediterranean Region [62], therefore species identification by molecular methods should be introduced to all endemic areas for better detection, diagnosis and well-directed treatment of CL.

The high percentage of infected household members (53.8%) in our sample and the detection of two brothers in Tarhuna infected with *L*. *tropica* (known as anthroponotic in many densely populated areas [66–68] indicated possible person-to-person transmission among households in Libya. The latter was identified as risk factor for CL and VL in many countries [33,69], hence screening of household members of CL patients should be considered for better surveillance of the disease. The life cycles of CL caused by different species of *Leishmania* in Libya need to be fully investigated since sandflies and rodents, or both, were frequently observed in the patient’s neighborhood. This will lead to a better understanding of disease dynamics and risk factors.

Pentostam is the first line treatment of CL in Libya. No significant changes in treatment regime and response were reported and treatment failure or resistance are not common. However, treatment unresponsiveness to Pentostam in a HIV co-infected Libyan CL patient was recently described [64]. Alternative therapy of such unresponsive cases, and when IM Pentostam is contra-indicated, includes combination of oral rifampicin (600 mg/day) and isoniazide (300 mg/day), thermotherapy and cryotherapy with or without intra-lesional Pentostam [64].

In 1991, the Dermatology Department in TCH was established to offer diagnostic and treatment services for leishmaniasis patients throughout Libya. Since then, many hospitals and clinics started to collaborate in the CL management at the national level. This included the establishment of 16 local leishmaniasis clinics by the LNCP in all over Libya which performed clinical diagnosis of CL and offered free treatment to all patients. Moreover, they were part of control and surveillance activities organised by LNCP and were involved in data collection and reporting of cases, spraying against sandfly vectors and control measures to contain putative reservoir hosts such as rodents and dogs. All leishmaniasis control activities offered by the LNCP clinics were suspended in 2011 due to the armed conflict. The latter hindered all efforts to introduce molecular diagnostic techniques for CL even to large cities in Libya, since traveling of experts from Libya for training abroad is intricate and recruitment of international experts is convoluted. Furthermore, all control and surveillance activities of CL during 2011–2012 had to be suspended due to the fact that most endemic areas were considered to be war zones and also due to the loss of equipment, infrastructures and vehicles owned by LNCP clinics. This situation has pushed many CL patients from the CL foci in northwest Libya to seek medical help in Tripoli Central Hospital TCH and other hospitals in more secure districts. The stock of Pentostam was adequate only in Tripoli hospitals and clinics. However, drug delivery to local leishmaniasis clinics in remote areas was completely suspended during 2011 until early 2012 when they partially reopened and started to work on part-time bases.

Moreover, massive movement of naïf population was seen during 2011–2012 for seeking secure or more peaceful regions. Migration and a reduced sanitary environment has increased the exposure to CL during this period and is considered as an accumulative risk factor. It should be emphasized that the reporting system of leishmaniasis in Libya is still interrupted and accurate information about disease spread and burden are not fully available.

## Supporting information

S1 Checklist STROBE checklist. (DOC)
