# Supplementary material for: Spatiotemporal and molecular epidemiology of cutaneous leishmaniasis in Libya
Source: PLoS Negl Trop Dis. 2017 Sep 7;11(9):e0005873. doi: 10.1371/journal.pntd.0005873 (PMC5605087; doi:10.1371/journal.pntd.0005873)
Supplement: S1 Checklist — (DOC) [file pntd.0005873.s001.doc]

STROBE Statement—Checklist of items that should be included in reports of ***cross-sectional studies***

|  | Item No | Recommendation |
| --- | --- | --- |
| **Title and abstract** | 1 | ‎✔ |
|  |  | ‎✔ |
| Introduction | | |
| Background/rationale | 2 | ‎✔ |
| Objectives | 3 | ‎✔ |
| Methods | | |
| Study design | 4 | ‎✔ |
| Setting | 5 | ‎✔ |
| Participants | 6 | ‎‎✔ |
| Variables | 7 | ✔ |
| Data sources/ measurement | 8* | ✔ |
| Bias | 9 | (N/A) |
| Study size | 10 | ✔ |
| Quantitative variables | 11 | ✔  If applicable, describe which groupings were chosen and why (N/A) |
| Statistical methods | 12 | (*a*) ✔ |
|  |  | (*b*) (N/A) |
|  |  | (*c*) (N/A) |
|  |  | (*d*) (N/A) |
|  |  | (*e*) (N/A) |
| Results | | |
| Participants | 13* | (a) ✔ |
|  |  | (b) (N/A) |
|  |  | (c) (N/A) |
| Descriptive data | 14* | (a) ✔ |
|  |  | (b) ✔ |
| Outcome data | 15* | ✔ |
| Main results | 16 | (*a*) (N/A) |
|  |  | (*b*) (N/A) |
|  |  | (*c*) (N/A) |
| Other analyses | 17 | (N/A) |
| Discussion | | |
| Key results | 18 | ✔ |
| Limitations | 19 | ✔ |
| Interpretation | 20 | ✔ |
| Generalisability | 21 | ✔ |
| Other information | | |
| Funding | 22 | ✔ |

*Give information separately for exposed and unexposed groups.

**Note:** An Explanation and Elaboration article discusses each checklist item and gives methodological background and published examples of transparent reporting. The STROBE checklist is best used in conjunction with this article (freely available on the Web sites of PLoS Medicine at http://www.plosmedicine.org/, Annals of Internal Medicine at http://www.annals.org/, and Epidemiology at http://www.epidem.com/). Information on the STROBE Initiative is available at www.strobe-statement.org.
